# Bridging the gap from welfare to education: Propensity score matching evaluation of a bridging intervention

**DOI:** 10.1371/journal.pone.0216200

**Published:** 2019-05-01

**Authors:** Michael Rosholm, Mai Bjørnskov Mikkelsen, Michael Svarer

**Affiliations:** 1 Department of Economics and Business Economics, Aarhus University, Aarhus, Denmark; 2 TrygFondens Centre for Child Research, Aarhus University, Aarhus, Denmark; 3 IZA Institute of Labor Economics, Bonn, Germany; 4 Department of Psychology, Aarhus University, Aarhus, Denmark; TED University, TURKEY

## Abstract

We evaluate a bridging intervention for a group of young people aged 18–29, with no formal educational qualifications, who are not in employment, education or training. The bridging intervention consisted of classroom training, educational internships and mentoring. Based on Danish register data with a large number of control variables, a propensity score matching estimator was applied to assess the effectiveness of the bridging intervention. The results show that the bridging intervention was effective in increasing educational enrollment and completion for all participants. The effects of the intervention were particularly large for participants assessed to be ‘not ready for education’ and those diagnosed with psychiatric disorders suggesting that the bridging intervention may be especially beneficial for these subgroups.

## Introduction

Although the majority of young people fare well in adulthood, acquire an education and become steady workers, a significant subgroup fails to do so. The number of young people aged 16–24 who are not in employment, education or training (NEET) is reported to be 23.4% in the EU, 15.5% in the USA, 12.2% in Australia, and 22.2% in the UK [1], and the global rate of NEET young people continues to increase [2]. In Denmark, the NEET rate is approximately 12% [1].

NEET young people represent a large economic burden for society and being a NEET has a negative impact at the individual level, manifesting as low social, physical and mental well-being [3–6]. Considering the costs to society as well as the negative individual consequences of being a NEET, there is a pressing need for effective interventions targeting NEET young people.

The existing evidence concerning interventions targeting disadvantaged and NEET young people tends to show weak effects on educational attainment, employment, unemployment, wages, and welfare receipt, and no effects on health measures [2,7–8]. For example, a recent comprehensive systematic review and meta-analysis reports an average effect size of 0.02 for interventions aimed at increasing employment rates for young people in high-income countries [8]. This significant but small effect size, suggests that more research is needed to develop effective interventions for NEET young people.

An extensive body of literature shows that the effectiveness of interventions aimed at promoting better adult outcomes for disadvantaged children and youth is highly age-dependent [9–11]. High-quality early-childhood interventions effectively improve life outcomes and show high economic returns, while interventions aimed at young adults are generally less effective and provide lower economic returns [12]. In addition, a recent review [7] indicates that the beneficial effect of interventions aimed at young children tend to persist long term, while interventions aimed at young adults appear to have few lasting benefits. As Cunha and Heckman [9, p. 5] conclude: *on average*, *the later remediation is given to a disadvantaged child*, *the less effective it is*.

Although past interventions to improve life outcomes for NEET young people have failed to provide lasting results, remediation for disadvantage may nevertheless be possible if the appropriate components are combined in interventions. For example, a few promising interventions have been documented in recent reviews of the literature [1,7,13]. These interventions primarily feature workplace-based interventions and mentoring. Workplace-based interventions integrate education with work, offer an opportunity to learn through experience, and promote character skill formation. Mentoring involves teaching character and social skills, scaffolding (i.e. tailoring interventions to the young adults’ needs), and surrogate parenting [12]. When integrating findings from these reviews, they appear to highlight similar key ingredients for successful interventions aimed at disadvantaged young people: a) integration of work and education, b) tailored and flexible interventions, c) high-contact, high-support interventions, where the individual is tracked closely, and d) financial incentives to participate. There is, however, still a paucity of evidence regarding the effectiveness of interventions designed to include these key ingredients.

In a Scandinavian welfare state context, the evidence is not encouraging either. A recent review and meta-analysis on the effectiveness of interventions aimed at NEET young people in the Nordic countries and a few other Northern European countries found large variability in the direction and size of effects, but concluded that the average effect is very small [14]. This mirrors findings in an analysis of Danish interventions aimed at NEET young people [15], and indicates that considerable public resources in the Northern European/Nordic welfare states may be wasted through substantial investments in ineffective interventions.

Taken together, the overall evidence for interventions targeting NEET young people is discouraging. The negligible effects of such interventions may reflect age-related declines in the ability to learn and change habits from young childhood to adolescence and early adulthood. Such developmental changes may make it more difficult for youth to benefit from remediating interventions. On the other hand, the pattern of findings from previous research may reflect lack of properly designed interventions for NEET young people; e.g. interventions that are implemented with high fidelity and include all or more of the key ingredients mentioned above.

The purpose of the present study was to investigate an intervention called ‘bridging’, aimed at increasing educational enrollment and completion for NEET young people. This intervention included key ingredients identified in the literature as being essential for successful interventions aimed at NEET young people. The intervention was implemented with high fidelity in a Danish context.

## Materials and methods

Here we briefly describe the context of the Danish educational system, the intervention, the treatment as usual, the sampling frame and data used for the analysis, the outcomes analyzed, and the statistical procedure used to estimate the effect of the intervention.

### Context: The Danish education system

The Danish state offers free education for all young people residing in Denmark. There are no tuition fees, and young people above the age of 18 are eligible for an educational grant at the amount of approximately $1000 per month (DKK 6.090 in 2018, taxable income).

Compulsory school consists of primary and lower secondary school and mostly takes place in public schools (16% of pupils in Denmark attend private schools, which are also heavily subsidized). Compulsory school consists of grades 0–9, and ends with an exam. It is not a requirement that the young person attends nor passes this exam, but in order to commence high school, passing it is required. No such requirement was present for entering vocational school at the time the intervention took place (a recent reform introduced stricter requirements to enter high school and vocational school, but this was implemented after the evaluation period used in this study and young persons in the bridging intervention were therefore not affected by the reform).

After compulsory school, there is a two-tier system with an academic track; high school, and a vocational track; vocational school. The vocational track provides formal educational qualifications that are recognized in the labour market (e.g., carpenter, auto mechanic, hair dresser, etc.), while the high school track qualifies you for further studies. In some cases, the vocational track may also qualify you for (specific) further studies. High school and vocational school are jointly labelled ‘youth education’.

High school lasts three years. The vocational programs are centralized at vocational schools, which students can attend after having completed compulsory school, i.e. typically from the ages 16–17 years. The vocational program consists of a basic track lasting one school year. After completing the basic track, students are required to obtain an apprenticeship at a company or in a vocational school facility in order to enter the main track of the vocational program. The main track consists of a sequence of internship periods and school periods (varying by the specific education). Conditional on the specific vocational education, the main track typically has a duration of 2 to 3.5 years. The entire vocational education thus lasts 3–4.5 years.

While in the basic track, students receive the educational grant. During the main track, the contracting firm pays the student a trainee wage. For those failing to find an internship firm, school-based internships (with student grants) are available.

NEET young people, who are not in employment, education or training, may receive social assistance, provided that they engage in activities aimed at preparing them for education or work. In May 2017, there were 169.400 NEETs in Denmark. Of these, about 36,360 (corresponding to 21.5%) were on social assistance [16]. Social assistance for young people below 25 is essentially at the same monthly level as the educational grant, while it is larger for those above 25.

### The “bridging” intervention

The intervention “Bridging the gap between welfare and education” (original Danish title: “Brobygning til uddannelse”) was initiated in 2013 by The Danish Agency for Labour Market and Recruitment in collaboration with the Danish Ministry of Education. A Danish policy report [17] describes the intervention in detail.

#### Target group and goal

The intervention was aimed at NEET young people aged 18–29, who received social assistance, were not enrolled in education and did not have a qualifying education. The goal was to support these young adults in getting ready to commence and complete a youth education. If this was not possible, a secondary goal was to help them find stable employment.

#### Recruitment

When a young person reports to the local jobcenter in order to receive social assistance, a dialogue is initiated with the aim of assessing the skills of the young person to evaluate whether and what type of education or employment that may be relevant for him or her. Based on the results of this evaluation, the young person is categorized as ‘ready for education’ or ‘not ready for education’. All young persons are then presented with the different opportunities for participating in various programs, of which ‘bridging’ is one. All young persons who report to the jobcenter to receive social assistance have to participate in some type of program, although the timing of program may vary across jobcenters and individuals depending on availability and local practice.

#### Implementation sites and period

The intervention was implemented at 12 different locations, geographically distributed all over Denmark, typically at a vocational school or some other youth education institution. The 12 sites were selected by The Danish Agency for Labour Market and Recruitment. The bridging intervention started in early March 2013 and enrolled participants until the end of 2014. The intervention lasted 14 weeks on average.

#### Intervention content

The intervention comprised an intensive 25-hour weekly schedule. It consisted of the following components:

1) Classroom training of cognitive skills (language and mathematics), social and character skills, and community participation.

2) Educational internships, where the young people visited different educational tracks at the school (and at other schools if needed), and job training, where they had the chance to try out different jobs.

3) Assistance from a mentor, who provided support with any personal and educational problems that the young people may have had. The mentor-mentee relationship continued into ordinary education and ended only when a formal internship was obtained.

For individuals who did not obtain the cognitive and socio-emotional skills necessary for completing an education, the intervention ended with the formulation of a ‘plan B’ for obtaining employment.

#### Procedure

A young person entered the bridging intervention after being offered access at a visitation meeting with a caseworker at the local jobcenter. The caseworker decided whether the young person was eligible for the intervention. If the young person was deemed eligible, they would start the bridging intervention as soon as possible. As explained above, participation in some type of program was a requirement for social assistance receipt, but the type of program was optional to the young person.

In a first step, an educational plan was formulated in collaboration between the young person, the jobcenter, and the educational institution. The plan was based on the abilities and desires of the young person. The young person was further assigned a contact person from the jobcenter, who would function as a mediator if complications arose during the course of the intervention. An emphasis was put on ensuring that the participant had only one contact person throughout the intervention. The young person was further assigned a mentor and screened for cognitive abilities (reading, and writing, mathematics). If the results of the screening warranted it, they were offered additional educational assistance. The bridging intervention took place in ‘real’ educational environments, where the participants could encounter other young persons engaged in education. The intervention included a fixed schedule with meaningful activities to accustom participants to the routine at an ordinary educational institution. These activities included classroom training, as well as educational and vocational internships. Outreach with the aim of supporting the young person in obtaining an internship was offered along with individualized support to obtain an internship. Absence implied an economic sanction corresponding to a day of social assistance.

For those that did not succeed or did not fit into the bridging intervention, an alternative course of action (Plan B) was formulated in collaboration with the young person.

#### Treatment as usual

Treatment as usual consisted of traditional, Danish activation measures, such as classroom and on-the-job-training, internships and subsidized employment programs. The majority of these interventions took place in ‘protected environments’ (e.g., so-called ‘production schools’) in contrast to the bridging intervention, which took place in real educational institutions.

### Sampling frame and data

During the intake period, a total of 2,726 young persons were registered as having participated in the intervention with a valid starting date (when more than one starting date was available, the earliest was used).

For each participant, we had a personal identifier for identification across several administrative registers. Still, 103 participants could not be tracked in the administrative registers, presumably because they were outside the relevant age range at the time of registration, or because their personal identifier was not typed correctly. Two hundred and eight individuals did not fulfill the requirements for the intervention as they were not receiving social assistance on the date of registration. Hence, the final sample consisted of 2,415 young individuals.

The data on the participants were transferred to the research servers of Statistics Denmark, who merged them with other administrative registers and anonymized the data such that there was still an (anonymized) identifier of the individual. This provided access to a rich set of background information. We had access to detailed data on educational enrolment and completion with starting and ending dates until the end of 2017, which was used to construct the main outcomes. Further, we had access to detailed weekly data on types of income transfers received and labour market history until the end of 2017. As such, we were able to follow participants at least 159 weeks and at most 252 weeks. About 70% of the participants could be followed for at least 200 weeks (approx. 4 years).

In addition to data on education and work status, we had access to background information such as age, gender, ethnicity, geographical location, whether or not they were registered as substance abusers (voluntary registration), whether or not they were placed in foster care or received preventive in-home interventions during childhood, and their net wealth (excluding wealth in pension funds and cash holdings) as of the end of 2012 or 2013 (depending on whether the date of entry into the intervention was in 2013 or 2014, respectively). Moreover, we had access to data on compulsory school leaving grades, subjective caseworker assessments, diagnoses for any physical diseases and psychiatric disorders registered during the three years before entry into the intervention, and criminal convictions since 2000.

The entire population aged 18–29 in 2013 in Denmark numbered 895,664 individuals. In order to generate a control group, we assigned each individual in the population a synthetic starting week as a random draw from a uniform distribution split into 93 equal length intervals, each corresponding to a starting week between week 10 of 2013 and week 50 of 2014, both included. After conditioning on the young person a) not having completed an education before the synthetically generated starting week, and b) being on social assistance in that week, we had a comparison sample of 54,339 individuals, who all fulfilled the formal requirements for participating in the bridging intervention, but did not participate. This comparison sample constituted our potential control group.

### Statistical analysis

To assess the effectiveness of the bridging intervention, we use a propensity score matching (PSM) estimator [18]. The PSM approach is often used in non-experimental settings, when the research team has access to rich background characteristics of both participants and a group of comparable non-participants. The basic idea of the PSM approach is that, after matching participants to potential controls on a function of observed characteristics (the propensity score, i.e., the estimated probability of participating in the intervention), any remaining *ex ante* differences between participants and matched controls are due to chance. The assumption that needs to hold for this estimator to be valid is a conditional independence assumption; that is, conditional on the included observed variables, the treatment and matched control groups are identical with respect to the expectation of the potential outcome(s) of interest, and hence, given this identifying assumption, the PSM allows for a causal interpretation of the *ex post* outcome differences between the treated and the matched control group as an average treatment effect on the treated.

The combination of a large potential control group and large set of conditioning variables strengthens the validity of the conditional independence assumption to circumvent the identification problem in evaluating the impact of the bridging intervention. In particular, access to rich data on labour market and education histories of the population is crucial in obtaining results not contaminated with selection bias [19–20].

The main results are based on a Nearest Neighbor matching procedure with 5 neighbors and with replacement. Balancing tests of all included variables between the treated and the matched control group are shown in Table A in S1 File. The tests reveal very small biases and show no significant differences on any conditioning variables *ex ante*.

All estimations were conducted using the PSMATCH2 procedure in STATA 15, where 95% confidence bands were obtained by bootstrapping the entire estimation process 200 times.

## Results

### Descriptive statistics

Table 1 presents descriptive statistics for the 2,415 individuals who participated in the bridging intervention as well as the 54,339 individuals who formed the control group.

**Table 1:** Descriptive statistics.

	Treatment group		Potential control group	
	Mean	SD	Mean	SD
**Enlisted in education in registration week:**				
Compulsory school	0.159		0.122	
High school	0.026		0.033	
Vocational school, basic track	0.108		0.052	
Vocational school, main track	0.058		0.040	
Further education	0.006		0.007	
**Socio-demographics**				
Male	0.595		0.505	
Married	0.043		0.080	
Age, years	22.84	31.24	25.05	42.98
1st or 2nd gen. western immigrant	0.016		0.026	
1st gen. non-western immigrant	0.062		0.113	
2nd gen. non-western immigrant	0.042		0.052	
Registered drug abuser	0.028		0.038	
Registered alcohol abuser	0.002		0.006	
In foster care during childhood	0.197		0.219	
Received preventive social intervention during childhood	0.283		0.280	
Net wealth, DKK	-23,278	80,199	-34,308	124,724
**Past history and subjective assessment by caseworkers**				
Finished high school	0.046		0.075	
Fraction of past three years on social assistence	0.405	0.321	0.565	0.343
Fraction of past three years in employment	0.098	0.164	0.076	0.156
Fraction of past three years with educational grant	0.213	0.244	0.129	0.222
CW: Not categorized	0.077		0.047	
CW: Not ready for education	0.365		0.541	
**Average grades from compulsory school**				
Danish school leaving exam	3.79	2.48	4.57	2.74
Danish teacher assessment	3.76	2.56	4.57	2.75
Math school leaving exam	3.32	2.78	3.79	2.99
Math teacher assessment	3.44	2.59	4.00	2.85
Missing danish school leaving exam	0.383		0.572	
Missing danish teacher assessment	0.364		0.556	
Missing math school leaving exam	0.394		0.582	
Missing math teacher assessment	0.366		0.559	
**Physical diagnoses in past three years**				
Cancer	0.003		0.004	
Diabetes	0.009		0.009	
Diseases of the nervous system	0.035		0.051	
Cardio-vascular diseases	0.015		0.028	
Respiratory diseases	0.046		0.053	
Musculoskeletal diseases	0.097		0.124	
Pregnancy and maternity related diseases	0.140		0.175	
Diseases of the digestive system	0.121		0.162	
Diagnostical examinations	0.463		0.493	
Accidents etc.	0.443		0.431	
Other diseases	0.399		0.463	
**Psychiatric diagnoses in the past three years**				
Organic, including symptomatic, mental disorders	0.002		0.005	
Mental and behavioural disorders due to psychoactive substance use	0.064		0.096	
Schizophrenia, schizotypal and delusional disorders	0.017		0.054	
Mood [affective] disorders	0.041		0.082	
Neurotic, stress-related and somatoform disorders	0.074		0.125	
Behavioural syndromes associated with physiological disturbances and physical factors	0.006		0.015	
Disorders of adult personality and behaviour	0.027		0.077	
Mental retardation	0.005		0.011	
Disorders of psychological development	0.014		0.024	
Behavioural and emotional disorders with onset usually occurring in childhood and adolescence	0.041		0.065	
Unspecified mental disorder	0.011		0.027	
**Criminal convictions**				
violent or sexual crimes	0.099		0.129	
Property crime	0.227		0.249	
Traffic crime	0.184		0.199	
Drug related crime	0.071		0.112	
Other crime	0.084		0.120	
**Municipality/jobcenter indicators**	Yes		Yes	
**Intervention starting week indicators**	Yes		Yes	
**N**	2,415		54,339	

Note: Means represent fractions unless otherwise noted.

Being registered as already enlisted in education in the week of registration would ideally not be possible, since students are not eligible for social assistance. However, while dates of entry into a certain education are considered quite precise, de-registration in case of drop-out or change of studies are not. Hence, some individuals may be on social assistance while still formally registered as being enlisted in an education. In addition, it *is* possible to take courses enabling you to complete compulsory school leaving exams while being on social assistance, and about 15% of the treated and 12% of potential controls did so. Very few were registered as being in high school, while 15% of the treated and 9% of potential controls were in vocational school. This difference was expected as vocational schools have drop-out rates around 20–25%, while drop-out from high school is much lower (around 15%).

Among the treated, almost 60% were men, and the average age in the sample was 23 years. There were 10% of non-western ethnicity, 20% were in foster care at some points during childhood, while 30% had received a preventive in-home intervention from the public child protection system during childhood. On average, they had negative net wealth (debts, mostly in the form of outstanding loans) corresponding to about one month’s wage in an unskilled occupation.

The treated group had spent on average 40% of the past three years on social assistance, 10% in employment, 20% in the educational system (with a study grant), and the remaining time in neither of these states. Slightly more than 1/3 of the sample was considered “not ready” to commence an education by their caseworker.

Close to 40% in the treated group had no school leaving exams in language or mathematics. Furthermore, they had fairly high rates of somatic diagnoses, psychiatric diagnoses and criminal convictions.

Compared to those in the potential control group, the treated group appeared overall to be slightly less disadvantaged.

### Estimation of the propensity score

The probability of participating in the intervention is estimated in a probit model on the sample of 2,415 participants in the bridging intervention as well as the 54,339 potential controls. The results are shown in Table 2.

**Table 2:** Probit model for participating in the bridging intervention.

	Coefficient	Std.err.	T	P-value
**Enlisted in education in registration week:**				
Compulsory school	-0.069	0.037	-1.850	0.064
High school	**-0.200**	**0.073**	**-2.730**	**0.006**
Vocational school, basic track	**0.243**	**0.043**	**5.590**	**0.000**
Vocational school, main track	0.079	0.055	1.420	0.155
Further education	-0.002	0.150	-0.010	0.991
**Socio-demographics**				
Male	**0.197**	**0.032**	**6.230**	**0.000**
Married	-0.107	0.057	-1.870	0.062
Age	**-0.064**	**0.005**	**-13.780**	**0.000**
Western immigrant or child of western immigrant	-0.130	0.091	-1.430	0.153
Non-western immigrant	**-0.101**	**0.049**	**-2.040**	**0.042**
Child of non-western immigrant	**-0.126**	**0.060**	**-2.110**	**0.035**
Registered drug abuser	0.070	0.074	0.950	0.344
Registered alcohol abuser	-0.309	0.238	-1.300	0.195
In foster care during childhood	0.044	0.034	1.300	0.194
Received preventive social intervention during childhood	0.003	0.030	0.090	0.925
Net wealth, 100.000 DKK	0.004	0.011	0.380	0.708
**Past history and subjective assessment by caseworkers**				
Finished high school	**-0.238**	**0.059**	**-4.030**	**0.000**
Fraction of past three years on social assistence	-0.055	0.057	-0.960	0.339
Fraction of past three years in employment	-0.157	0.087	-1.800	0.072
Fraction of past three years with educational grant	**0.406**	**0.065**	**6.260**	**0.000**
CW: Not categorized	**-0.112**	**0.055**	**-2.020**	**0.043**
CW: Not ready for education	**-0.380**	**0.028**	**-13.330**	**0.000**
**Average grades from compulsory school**				
Danish, school leaving exam	**-0.025**	**0.008**	**-3.290**	**0.001**
Danish, teacher assessment	**-0.026**	**0.008**	**-3.350**	**0.001**
Math, school leaving exam	-0.009	0.009	-1.050	0.295
Math, teacher assessment	**-0.019**	**0.009**	**-2.020**	**0.043**
Missing Danish grade, school leaving exam	-0.134	0.072	-1.850	0.064
Missing Danish grade, teacher assessment	-0.116	0.091	-1.260	0.206
Missing math grade, school leaving exam	**-0.142**	**0.068**	**-2.080**	**0.038**
Missing math grade, teacher assessment	-0.121	0.091	-1.330	0.182
**Physical diagnoses in past three years**				
Cancer	0.044	0.213	0.210	0.837
Diabetes	0.039	0.133	0.290	0.769
Diseases of the nervous system	-0.071	0.065	-1.090	0.278
Cardio-vascular diseases	**-0.217**	**0.094**	**-2.300**	**0.021**
Respiratory diseases	-0.016	0.058	-0.280	0.778
Musculoskeletal diseases	**-0.083**	**0.041**	**-2.010**	**0.045**
Pregnancy and maternity related diseases	0.060	0.044	1.360	0.174
Diseases of the digestive system	-0.046	0.039	-1.190	0.235
Diagnostical examinations	-0.011	0.028	-0.390	0.700
Accidents etc.	0.023	0.027	0.850	0.393
Other diseases	-0.028	0.031	-0.880	0.377
**Psychiatric diagnoses in the past three years**				
Organic, including symptomatic, mental disorders	-0.099	0.245	-0.400	0.687
Mental and behavioural disorders due to psychoactive substance use	-0.014	0.051	-0.280	0.778
Schizophrenia, schizotypal and delusional disorders	**-0.414**	**0.082**	**-5.070**	**0.000**
Mood [affective] disorders	0.007	0.060	0.110	0.909
Neurotic, stress-related and somatoform disorders	-0.043	0.047	-0.910	0.362
Behavioural syndromes associated with physiological disturbances and physical factors	-0.018	0.139	-0.130	0.895
Disorders of adult personality and behaviour	**-0.205**	**0.069**	**-2.950**	**0.003**
Mental retardation	-0.217	0.153	-1.420	0.155
Disorders of psychological development	**-0.239**	**0.099**	**-2.420**	**0.016**
Behavioural and emotional disorders with onset usually occurring in childhood and adolescence	-0.117	0.062	-1.870	0.061
Unspecified mental disorder	-0.167	0.101	-1.660	0.098
**Criminal convictions**				
violent or sexual crimes	-0.077	0.045	-1.710	0.087
Property crime	**0.082**	**0.034**	**2.420**	**0.016**
Traffic crime	0.036	0.036	0.990	0.321
Drug related crime	**-0.103**	**0.050**	**-2.040**	**0.041**
Other crime	-0.069	0.048	-1.460	0.144
**Municipality/jobcenter fixed effects**	Yes		Yes	
**Intervention starting week fixed effects**	Yes		Yes	

Note: Coefficients in bold were significant at p < .05.

The results reveal that those registered as being enrolled in high school or had already completed high school were less likely to participate in the bridging intervention, while those enrolled in vocational schools were more likely to participate. Men were more likely to participate than women, and those who were older were less likely to participate than the young in the group. Immigrants and children of immigrants were less likely to participate than young persons born in Denmark to Danish-born parents.

Those with more experience in the educational system (as measured by the amount of time spent with an educational grant) had a higher probability of participating. Those categorized by their caseworker as being not ready for education had a lower participation propensity than those who were considered ready.

Those with lower school leaving exam grades were less likely to participate in the bridging intervention, as were those with cardiovascular or musculoskeletal diseases compared to without a diagnoses. Those with schizophrenia, personality disorders and mental development disorders were also less likely to take part in the bridging intervention compared to psychiatric diagnoses. Those who had been convicted of property crime were more likely to participate compared to those with no convictions, while those convicted of drug related crimes were less likely to participate.

Figure A in S1 File shows histograms of the propensity scores for the treatment and the potential control group. Only 10 treated persons are removed due to lack of common support. Hence, the impact estimates are based on 2,405 participants out of the 2,415 in the treated sample.

### Implementation rate

**Fig 1:**
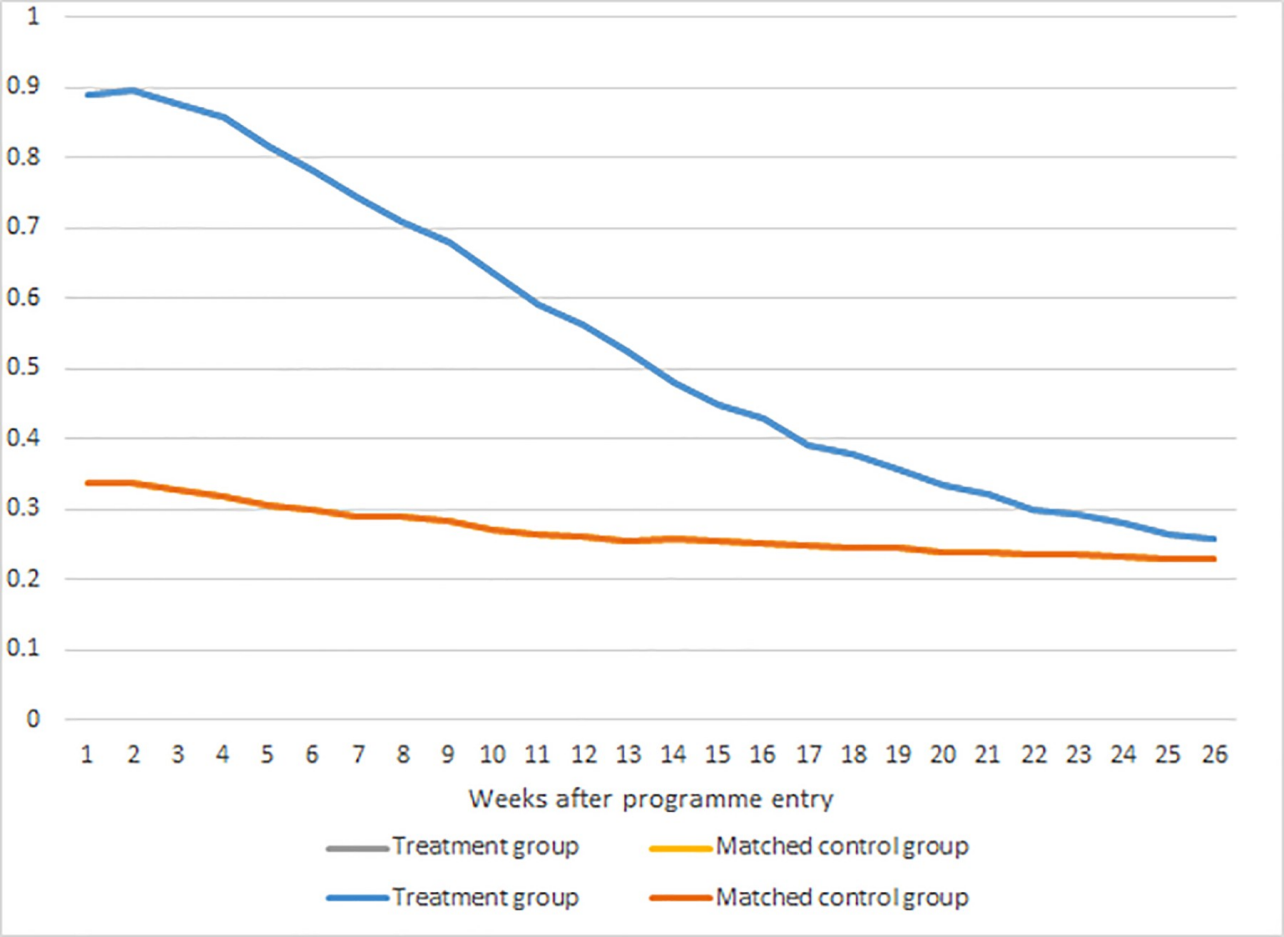
Program participation rates among the treated and matched controls.

The average rate of participation in some type of activity (bridging, ordinary active labour market programs, production schools etc.) in the treatment group and the matched control group is shown in Fig 1. In the group of treated, 90% were registered as intervention participants in the first week of treatment, implying that 10% of those assigned to the bridging intervention never showed up. Over time, the fraction in treatment declined, such that after 13 weeks 55% were still in the intervention, and after 26 weeks the fraction had decreased to slightly above 25%. In the matched control group, 35% were in some form of program in the assignment week, which then dropped to 25% after 26 weeks. Hence, there is not only a difference in the type of program in which the treated and the matched controls participated (bridging vs other programs), there was also a difference in the rate of participation, as should be expected. It is important to note that ‘treatment as usual’ refers to *different* treatment at a lower rate and not *absence of treatment* as described in Materials and Methods section.

### Overall effects

Below follows a set of figures illustrating the effect of participation in the bridging intervention on the likelihood of being enrolled in or completing various tracks in the educational system. Specifically, a distinction was made between completing the basic track of vocational school, the main track of vocational school, high school, and further education. Recall that the evaluation time period was relatively short (slightly below four years). Hence, it was not expected that all those in the treatment and matched control group would manage to complete the main vocational track, high school or further education; as already mentioned, the full (basic plus main) vocational track lasts 3–4.5 years, high school lasts 3 years, and further education requires high school completion and then lasts an additional 3–5 years. Moreover, most of the participants did not enter an education immediately after the start of the observation period.

In addition, we also looked at completing compulsory school leaving exams, since many of the participants (almost 40%) had not completed compulsory school leaving exam at the time of inclusion.

An individual is registered as enrolled in education if the individual in a given week is receiving the government funded student allowance (all students aged 18 and above who are enrolled in education beyond lower secondary school are eligible for these grants and take-up is close to 100%).

**Fig 2:**
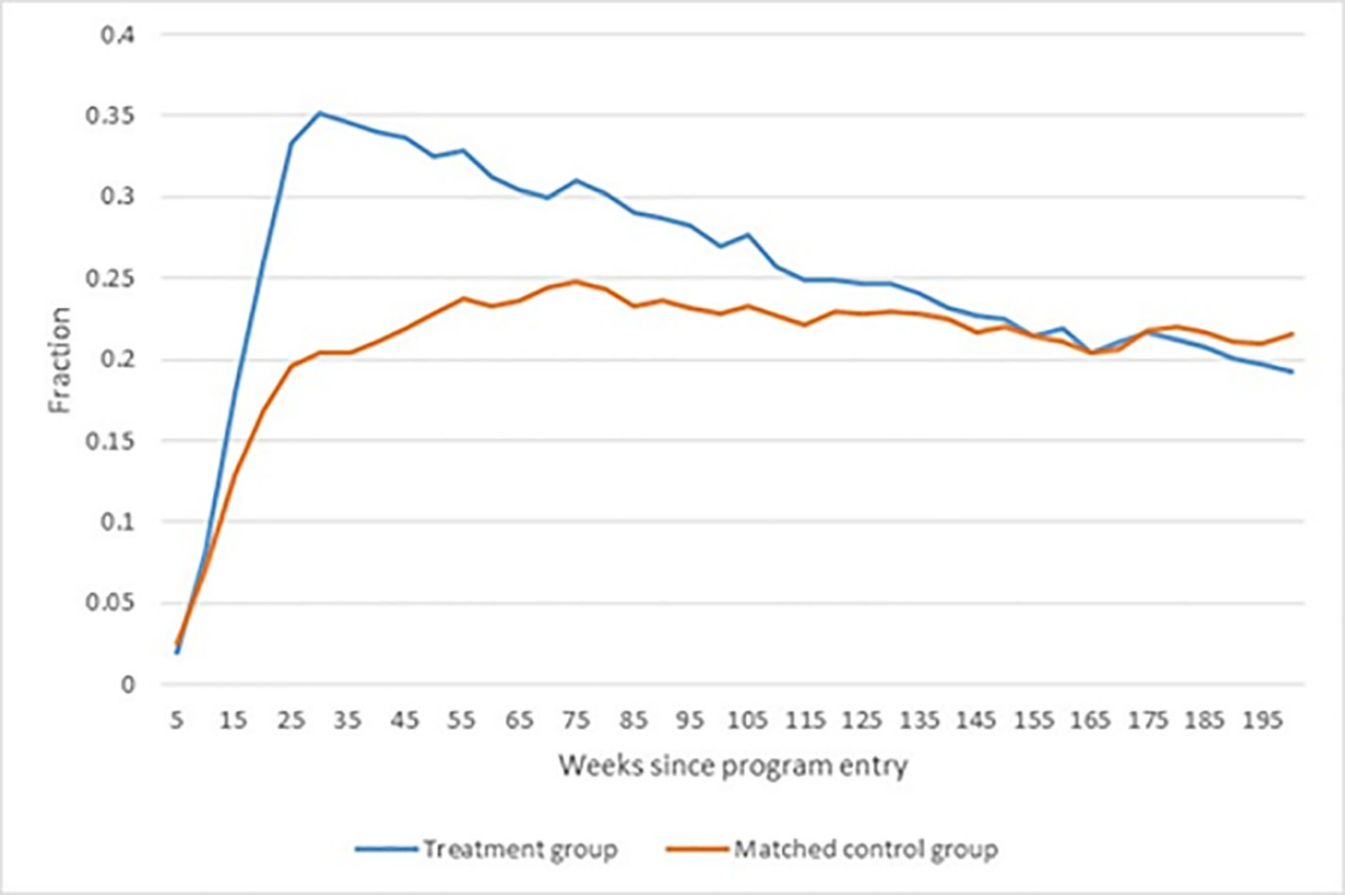
Fraction enrolled in education.

Fig 2 shows that participants entered and were enrolled in education to a significantly larger extent than the matched control group. Ten weeks into the bridging intervention, the enrolment rate among the participants accelerated compared to the enrolment rate for the matched control group and reached a peak 26 weeks after intervention start. At its peak, close to 35% of the participants were enrolled in some type of educational activity. The fraction enrolled in education decreased after 26 weeks, and at the end of the observation period, the fraction enrolled in education had converged in the two groups. The drop in enrolment for the treatment group may reflect that participants entered the main track in a vocational school and therefore would have received apprenticeship wages from their employer rather than the educational grant.

**Fig 3:**
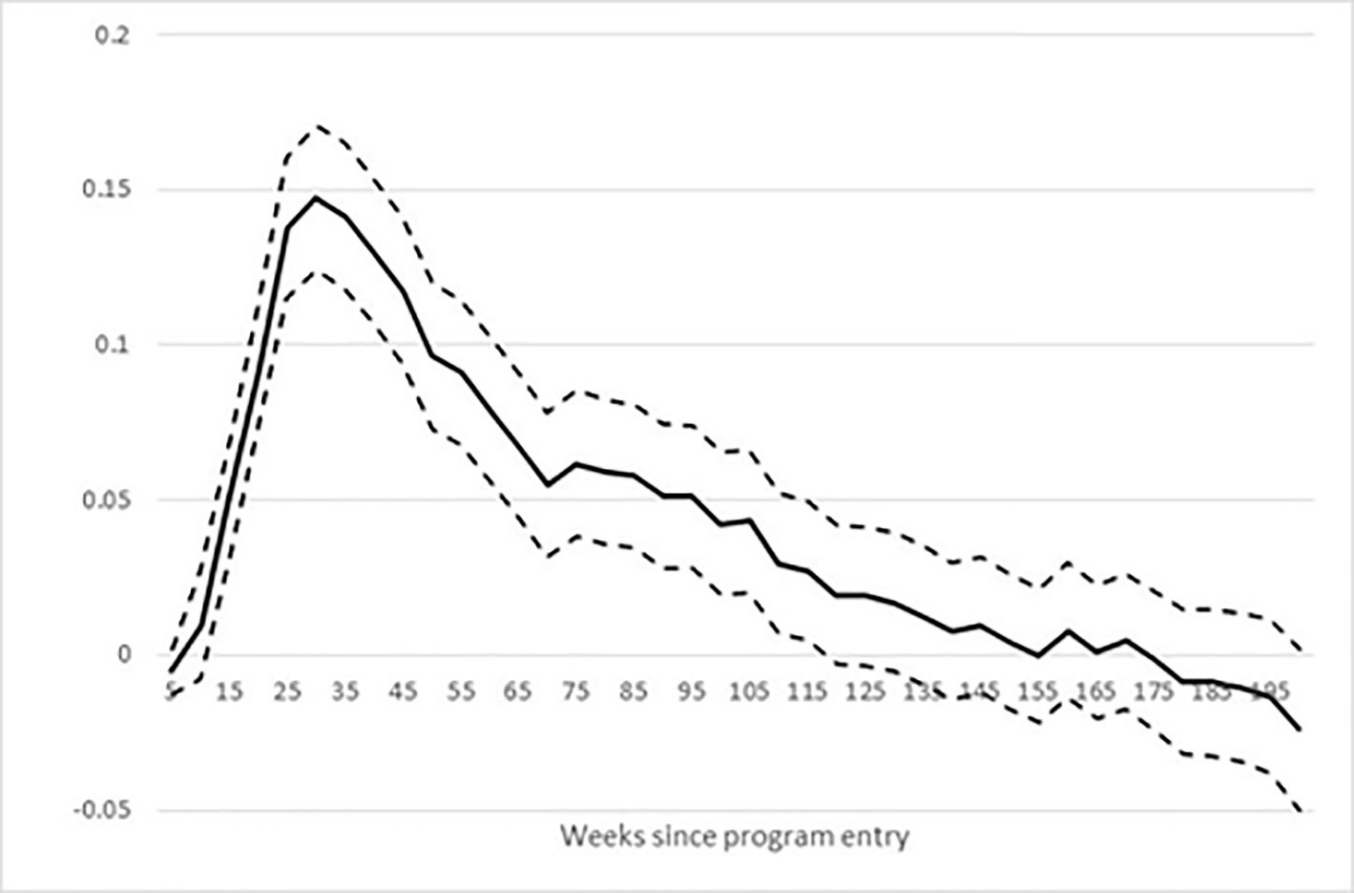
Effect of bridging intervention on enrolment in education. Note: Dotted lines show the 95% confidence band.

Fig 3 shows the effect of the bridging intervention on educational enrolment based on the preferred matching estimator and a 95% confidence interval.

Fig 3 shows that the effect of bridging on enrolment in any education is statistically significant. The enrolment rate nearly doubles at the peak level after half a year for the treated relative to the matched control group indicating that the intervention was effective in terms of enrolment into education.

**Fig 4:**
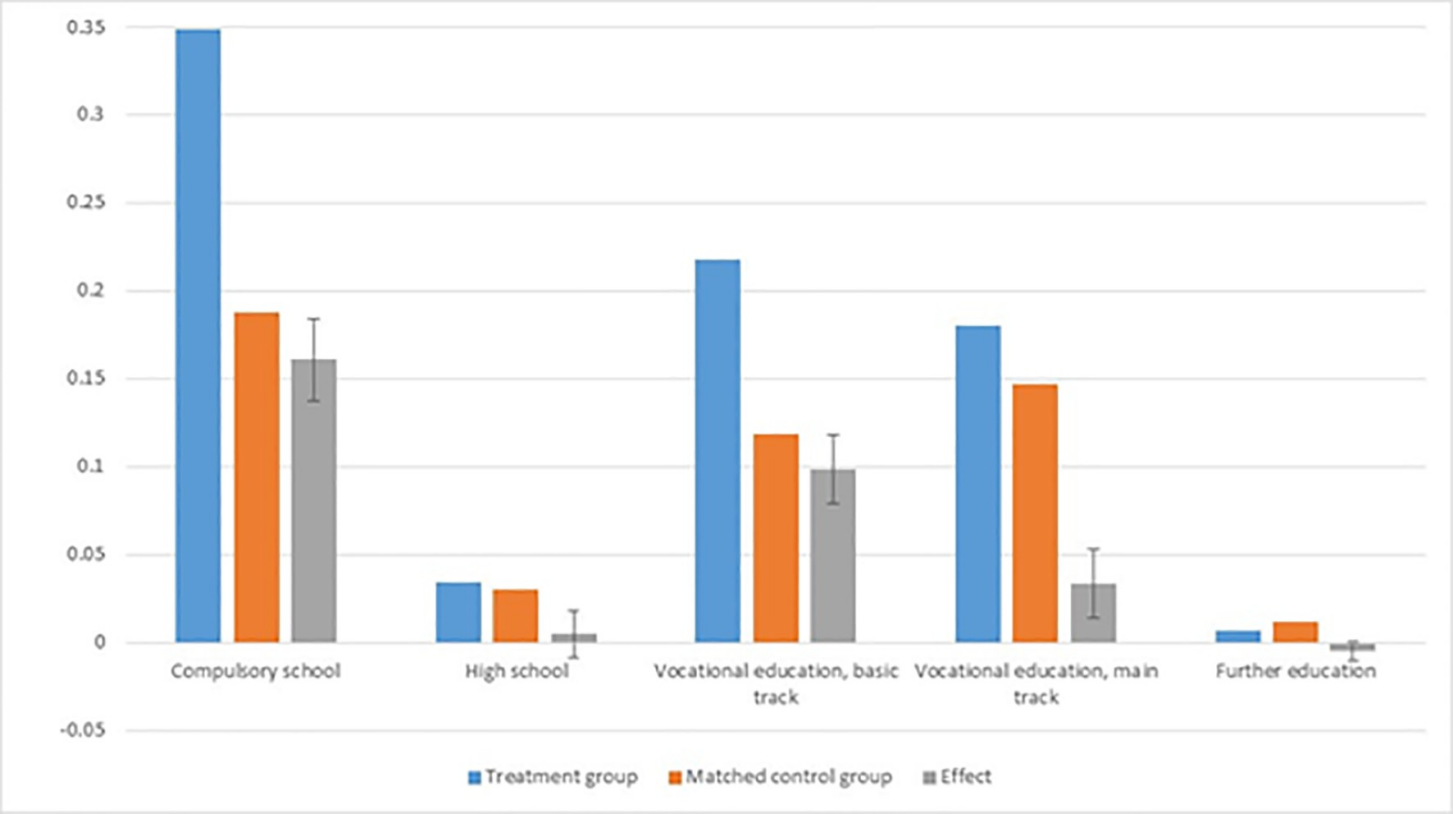
Effect on completed educational steps. Note: Vertical black lines are 95% confidence intervals.

Fig 4 shows the fraction of individuals that completed various levels of education for the treatment group (blue bars), for the matched control group (brown bars), as well as the effect of the intervention on educational completion (grey bars). The vertical black lines are 95% confidence intervals on the estimated effects.

As evident from Fig 4, the bridging intervention had a significant positive impact on completion of both compulsory school and the basic and main tracks of the vocational education system. Almost twice as many in the treatment group completed compulsory school (16%-point effect) and/or a basic track in the vocational schooling system (10%-point effect) compared to the matched control group. There was a 3.3%-point increase in the fraction completing the entire vocational education (main track). There was no impact on the likelihood of completing high school or further education.

**Fig 5:**
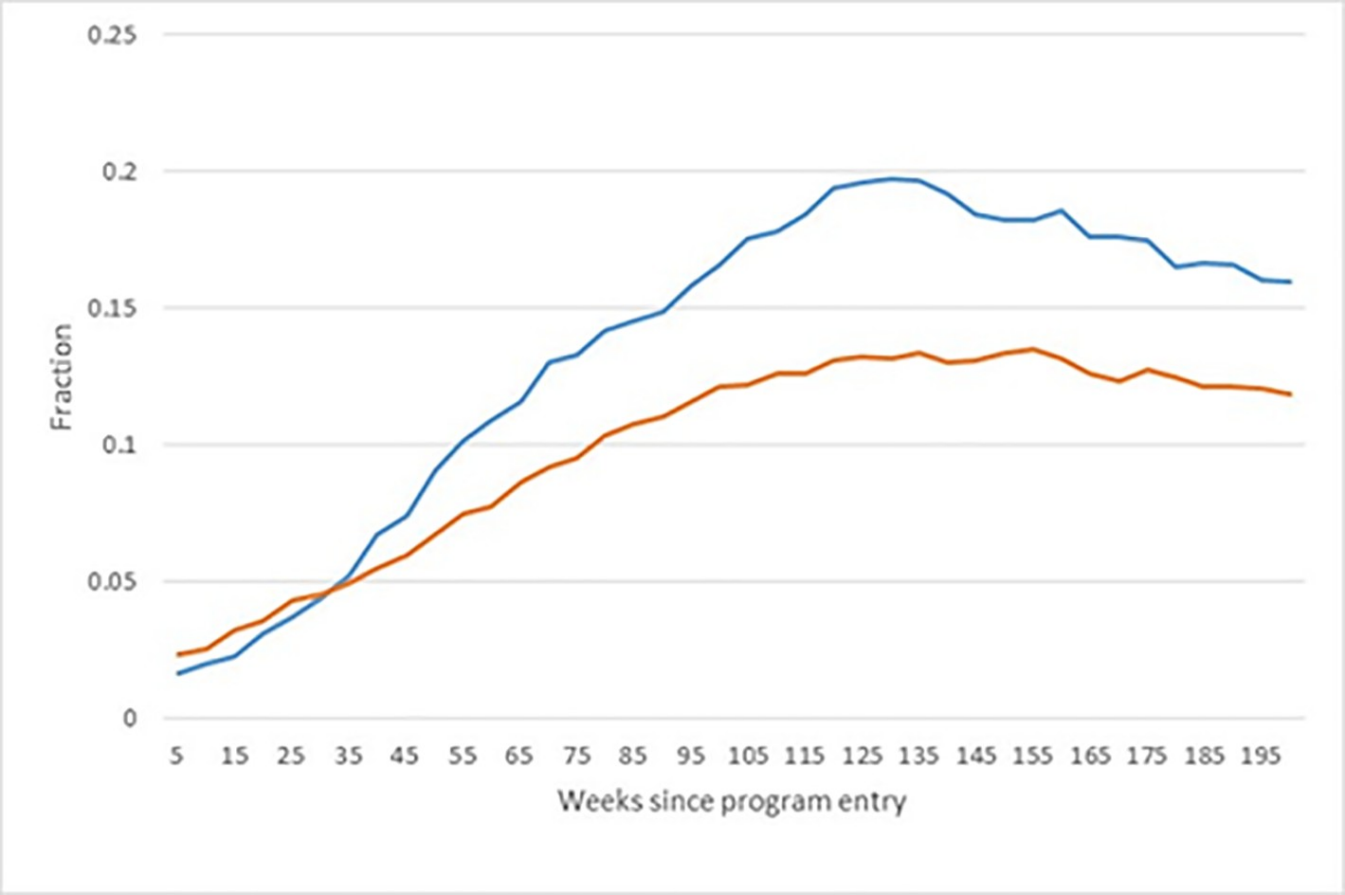
Fraction enrolled in the vocational main track.

**Fig 6:**
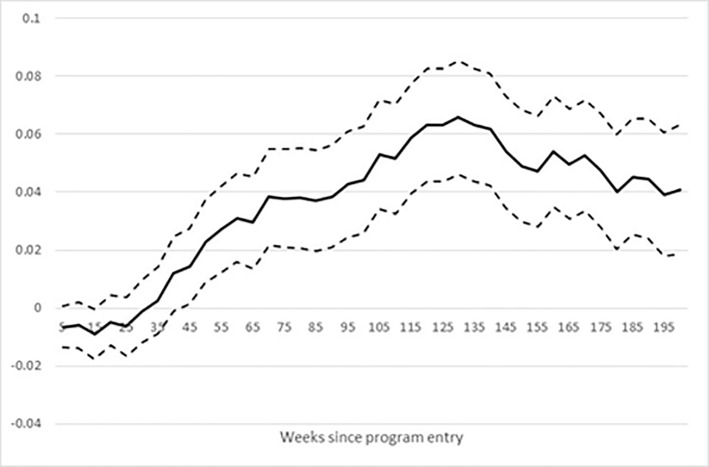
Effect on enrolment rate in vocational main track. Note: Dotted lines show a 95% confidence band.

To investigate the extent to which the effects on the completion of the vocational main track may be under-estimated due to the relatively short evaluation period, Figs 5 and 6 shows the enrolment rates in the vocational main track for the treatment group and the matched control group, and the impacts on enrolment, respectively.

Figs 5 and 6 show that a larger fraction of the treatment group was enrolled in a vocational main track compared to the matched control group. In addition, the difference between the groups was statistically significant from about 1 year after starting the bridging intervention. The drop in the effect on the enrolment rate in the main vocational track from around week 130 corresponds closely to the impact of the intervention on the fraction completing the main vocational track. Given that there was a 4%-point difference in enrolment rates at the end of the evaluation period, we may expect the impact on completed main vocational tracks to increase to 7–8% points, as there is little drop-out during the main track.

**Fig 7:**
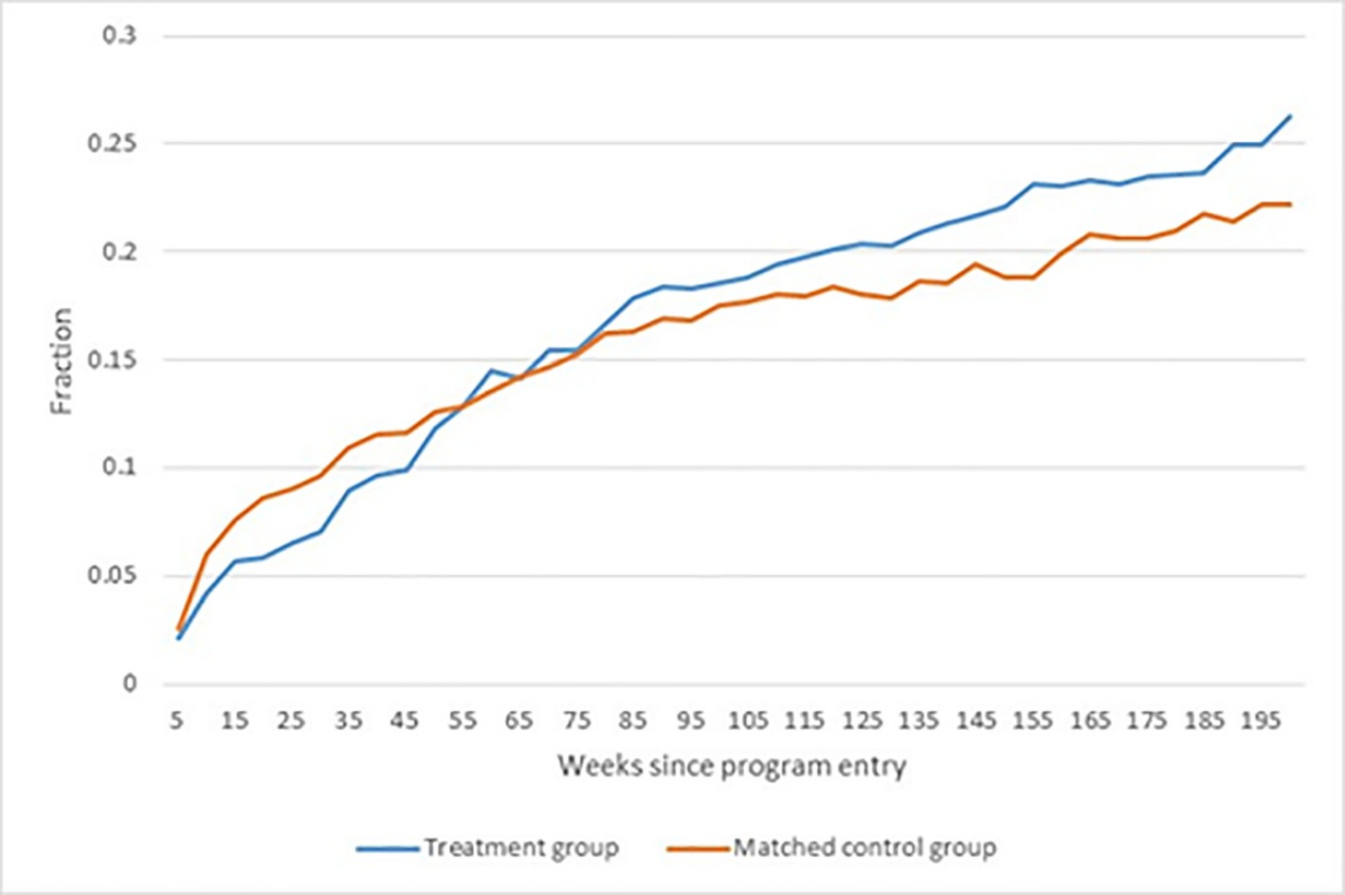
Fraction in employment.

**Fig 8:**
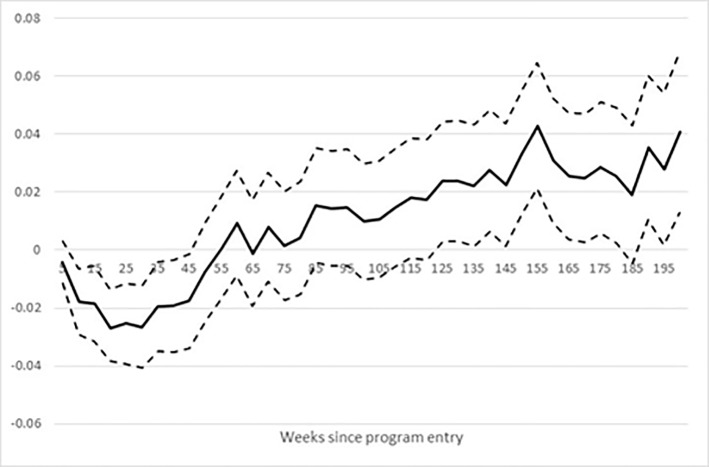
Effect of bridging on fraction in employment. Note: Dotted lines show a 95% confidence band.

Fig 7 shows the fraction in employment and Fig 8 the impact on the employment rate.

As shown in Fig 8, the bridging intervention had a negative impact on employment in the first year after the start of the intervention. This is most likely due to a lock-in effect of the intervention; those that enrolled in the bridging intervention may have searched less actively for employment because they wanted to complete the intervention and enter the education system. About a year after intervention start, the treatment group were more likely to be employed than the matched control group, but the difference only became statistically significant after about 2.5 years (125 weeks).

#### Sensitivity of main results

Sensitivity of the main results with respect to a variety of matching estimators is shown in Figure B in S1 File. The results show variation in the impacts on completion of the basic and main tracks in vocational school according to choice of matching technology. We vary the number of neighbors used for matching, using 1 (with and without replacement), 5 and 10 nearest neighbors. We also show results from kernel matching and local linear regression matching. A simple linear regression model based on the same set of characteristics is also included. As evident from the Figure B in S1 File, results are highly robust to the choice of matching estimator.

### Sub-group effects

Sub-group effects are only reported for completion of the basic vocational track, as we see completion of the basic track as a central indicator for completion of the main track, cf. the discussion in Overall Results section.

**Fig 9:**
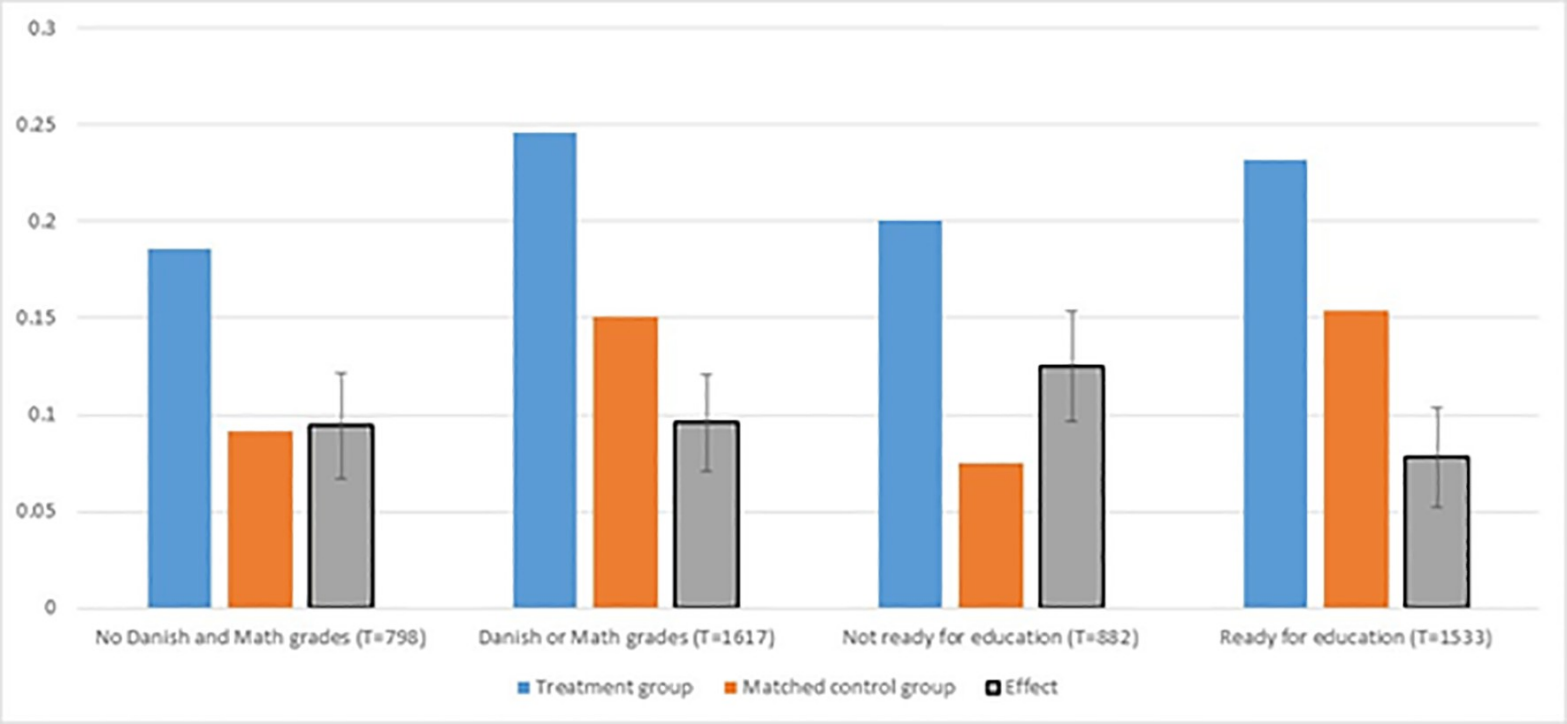
Sub-group impacts on completion of basic vocational track. Note: Vertical black lines are 95% confidence intervals.

We tested for sub-group effects in a number of dimensions, including gender, age, and ethnicity but did not find remarkable differences in those dimension. Fig 9 shows the impact of the bridging intervention on completion of the basic vocational track for two sets of sub groups; those with and without school leaving grades in Danish and mathematics, and those assessed to be ‘ready for education’ and ‘not ready for education’ by their case worker.

Note that impacts are of about the same magnitude, irrespective of whether the young person has taken parts of the compulsory school leaving exam (Danish or math grades) or not. Moreover, impacts tend to be larger for those evaluated *ex ante* by their caseworker to be ‘not ready for education’ when compared to those who were evaluated to be ‘ready for education’.

**Fig 10:**
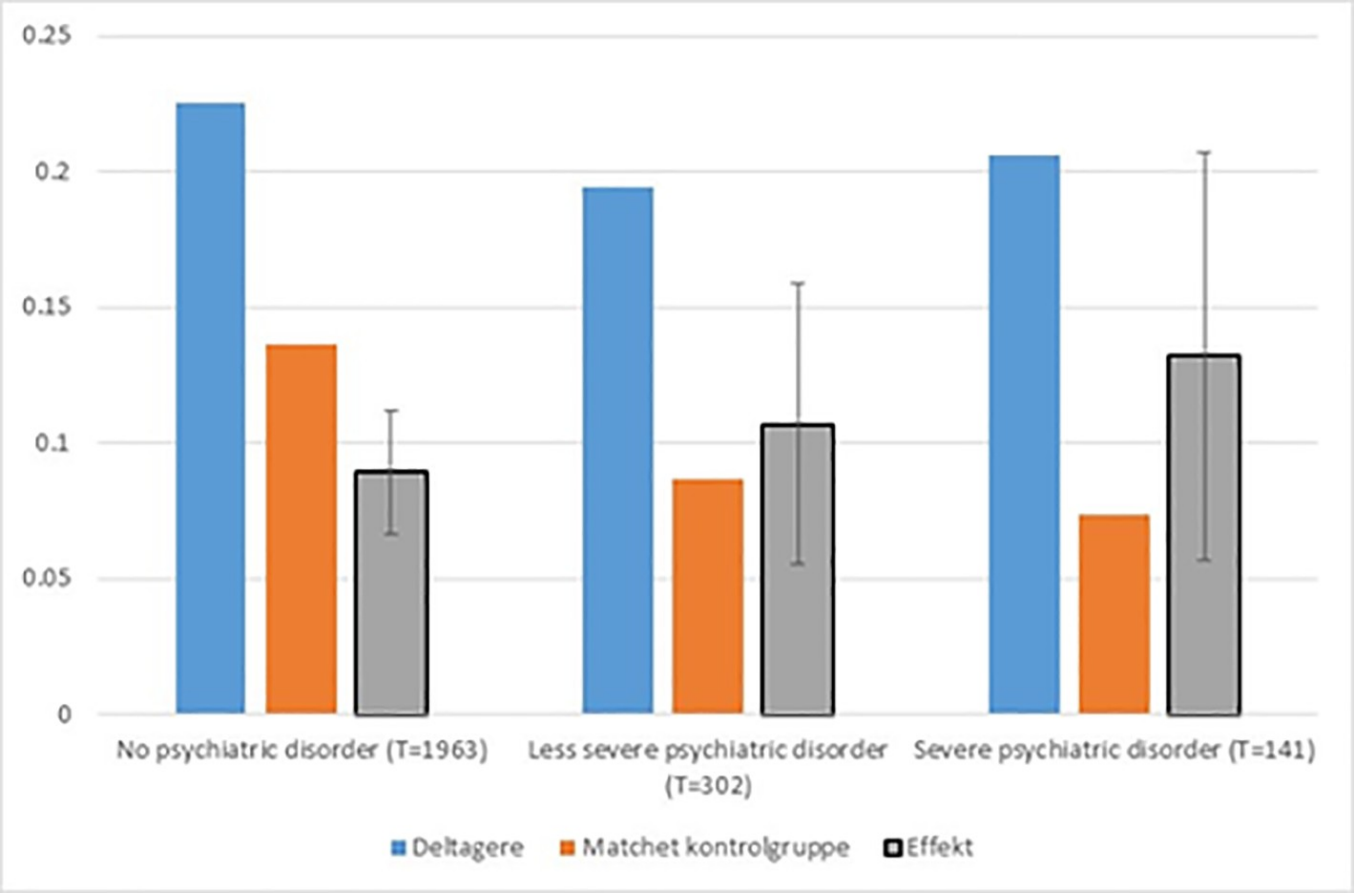
Impacts on completion of basic vocational track by severity of psychiatric disorder. Note: Vertical black lines are 95% confidence intervals.

Fig 10 shows impacts for sub groups defined by whether or not they had a psychiatric disorder and if so, by its severity.

Note that in the matched control group, the likelihood of completing the basic track diminishes with the severity of the psychiatric disorder, while the opposite pattern can be observed for the treatment group. Note, however, that none of the sub-group differences are statistically significant.

## Discussion and limitations

Around 15% of Danish young people do not obtain a qualifying education after leaving compulsory school. Many of these young people are characterized as NEET young people and end up in prolonged public income support. There is strong policy focus on NEETs in Denmark as well as at the EU level, and policies directed at assisting them in achieving education and work are plentiful. Neither in Denmark [21] nor internationally [8] has traditional active labour market polices proven effective for this particular group of NEET young people. These young people often struggle with poor mental and physical health, poor academic skills, low self-esteem, low self-control and are more often drug abusers, criminal, obese, and have financial problems [22–23]. Still, some scholars argue that interventions, which are intensive and directed towards enhancing the personal and social skills of the young persons, can have positive effects [12]. This is particularly the case if interventions include mentors/surrogate parenting, counseling and job training. In addition, programs that closely track and support the young people in various dimensions and address the problems they face (scaffolding) can have positive effects [7, 24–26].

The results of the present evaluation of the bridging intervention indicate that an intervention based in vocational schools and consisting of a combination of classroom training with focus on both cognitive and character skill formation, on educational internships, on job training in firms as well as mentoring produces significant effects on educational enrolment and completion for NEET young people. Moreover, contrary to many other intervention studies, the results suggest that this intervention is more effective for more disadvantaged groups such as those assessed to be ‘not ready for education’ and those with severe mental disorders, i.e. the more disadvantaged sub-groups. In addition, we did not find any remarkable differences in effect size across age, as found in other studies [9–11].

It should be noted that the estimated effects were based on 90% turn-up in the first week with subsequent decline. Moreover, the treatment as usual was not ‘no treatment’, but rather treatment of 35% of the matched control group in alternative programs. Hence, the estimated impacts are potentially a lower bound on the true effects for someone participating full time when compared to a non-participant who do not participate in any program.

**Effect size and costs**: In order to compare the effect of the bridging intervention to other interventions, we calculated standardized effect sizes as Glass’ delta [27] where the treatment effect on a given outcome is divided by the standard deviation of the same outcome in the (matched) control group. This leads to an effect size of 0.31 for completing the basic vocational track and 0.10 for completing the main vocational track. These effects are large in an international comparison, when it comes to interventions aimed at NEET young people; a systematic review [2] found effects on education outcomes that were on average smaller than those produced by the bridging intervention. A meta-analysis [8] found average effect sizes of 0.05–0.13 for various employment related outcomes of disadvantaged youth. The extra costs of the intervention (calculated as the total costs per individual minus the average costs of treatment as usual) was DKK15,648, corresponding to approximately USD2,500. This leads to an effect size per USD1,000 invested of 0.12, which is relatively large compared to results reported in other evaluations of interventions aimed at disadvantaged young persons (e.g., Chicago high school intervention, an earned income tax credit intervention, a class size reduction, and the Perry Preschool program; [25]). Hence, the bridging intervention is cost-effective and has large absolute effects on medium term educational outcomes for disadvantaged young people.

**Bias:** The analyses are based on a propensity score matching estimation, and therefore the results are based on the conditional independence assumption that potential outcomes in the non-treated state are identical in the treated and matched control group. As shown, the results are not sensitive to the choice of matching estimator, nor are they sensitive to whether or not a set of municipality-fixed effects are included in the estimation of the propensity score (not shown). Across participating municipalities, the fraction of NEET young persons on social assistance participating in bridging varied from less than two percent to almost 25 percent. The recruitment process described in Materials and methods implies that selection into bridging is not entirely voluntary, as the young persons have to participate in some form of program regularly. Taken together with the considerable (to a large extent exogenous) variation in treatment rates across municipalities, this implies that selection bias may not be a large concern. However, despite the rich set of conditioning covariates, including full educational and labour market histories, detailed grades, detailed health information, crime history, etc., there may still be unobserved differences between the treatment group and the matched control group that is not accounted for, and could bias the results.

**Time frame:** Although the investigation followed the participants for almost 4 years, longer term outcomes are crucial for evaluating whether the bridging intervention achieved the goal of redirecting NEET young people away from long-term dependence on public income transfers.

**External validity:** We evaluated the impact of treatment on the treated in the context of a Scandinavian welfare state. Hence, our results may not be valid for other groups of young people, nor may they be valid in different contexts.

## Conclusion

We evaluated the effects of the “bridging” intervention, an intervention aimed at a group of NEET young people between 18 and 29 years of age receiving social assistance. The bridging intervention consisted of class-room training, educational internships and mentoring. Based on Danish register-based data set with a large set of controls variables a propensity score matching estimator was applied to assess the effectiveness of the intervention. The results showed that bridging was successful in increasing educational enrollment and completion of qualifying education. In particular, bridging tended to show larger effects for those with severe mental diagnoses and those assessed to be ‘not ready for education’, i.e. the more disadvantaged groups.

## Supporting information

S1 File (DOCX)
