# Supplementary material for: Bridging the gap from welfare to education: Propensity score matching evaluation of a bridging intervention
Source: PLoS One. 2019 May 1;14(5):e0216200. doi: 10.1371/journal.pone.0216200 (PMC6494054; doi:10.1371/journal.pone.0216200)
Supplement: S1 File — (DOCX) [file pone.0216200.s001.docx]

# Supporting Information

**Table A.** **Balancing tests.**

|  | Mean, treatment group | Mean, mat-ched control group | % bias | T test | P value |
| --- | --- | --- | --- | --- | --- |
| **Enlisted in education in registration week:** |  |  |  |  |  |
| Compulsory school | 0.159 | 0.154 | 1.6 | 0.52 | 0.61 |
| High school | 0.026 | 0.026 | 0.1 | 0.04 | 0.97 |
| Vocational school, basic track | 0.107 | 0.113 | -2.4 | -0.73 | 0.47 |
| Vocational school, main track | 0.058 | 0.056 | 1.0 | 0.34 | 0.74 |
| Further education | 0.006 | 0.006 | 0.6 | 0.22 | 0.82 |
| **Socio-demographics** |  |  |  |  |  |
| Male | 0.593 | 0.591 | 0.5 | 0.18 | 0.86 |
| Married | 0.044 | 0.041 | 1.3 | 0.55 | 0.59 |
| Age | 22.851 | 22.738 | 3.0 | 1.18 | 0.24 |
| 1st or 2nd gen. western immigrant | 0.015 | 0.017 | -0.9 | -0.37 | 0.71 |
| 1st gen. non-western immigrant | 0.062 | 0.059 | 0.9 | 0.36 | 0.72 |
| 2nd gen. non-western immigrant | 0.042 | 0.047 | -2.4 | -0.87 | 0.39 |
| Registered drug abuser | 0.028 | 0.028 | -0.3 | -0.12 | 0.90 |
| Registered alcohol abuser | 0.002 | 0.002 | 0.0 | 0.00 | 1.00 |
| In foster care during childhood | 0.197 | 0.198 | -0.1 | -0.04 | 0.97 |
| Received preventive social intervention during childhood | 0.284 | 0.282 | 0.2 | 0.08 | 0.93 |
| Net wealth, DKK | -23,313 | -19,881 | -3.2 | -0.90 | 0.37 |
| **Past history and subjective assessment by caseworkers** |  |  |  |  |  |
| Finished high school | 0.046 | 0.044 | 0.6 | 0.22 | 0.82 |
| Fraction of past three years on social assistence | 0.406 | 0.404 | 0.8 | 0.27 | 0.79 |
| Fraction of past three years in employment | 0.098 | 0.100 | -1.2 | -0.39 | 0.70 |
| Fraction of past three years with educational grant | 0.212 | 0.217 | -2.1 | -0.65 | 0.51 |
| CW: Not categorized | 0.077 | 0.078 | -0.2 | -0.06 | 0.95 |
| CW: Not ready for education | 0.366 | 0.376 | -2.2 | -0.76 | 0.45 |
| **Average grades from compulsory school** |  |  |  |  |  |
| Danish school leaving exam | 2.342 | 2.408 | -2.4 | -0.84 | 0.40 |
| Danish teacher assessment | 2.394 | 2.467 | -2.6 | -0.91 | 0.36 |
| Math school leaving exam | 2.007 | 2.055 | -1.8 | -0.62 | 0.54 |
| Math teacher assessment | 2.179 | 2.243 | -2.4 | -0.83 | 0.41 |
| Missing danish school leaving exam | 0.384 | 0.374 | 2.0 | 0.71 | 0.48 |
| Missing danish teacher assessment | 0.365 | 0.355 | 2.2 | 0.77 | 0.44 |
| Missing math school leaving exam | 0.395 | 0.383 | 2.6 | 0.89 | 0.37 |
| Missing math teacher assessment | 0.368 | 0.358 | 2.1 | 0.76 | 0.45 |
| **Physical diagnoses in past three years** |  |  |  |  |  |
| Cancer | 0.003 | 0.003 | 0.4 | 0.16 | 0.87 |
| Diabetes | 0.009 | 0.007 | 1.4 | 0.52 | 0.61 |
| Diseases of the nervous system | 0.035 | 0.036 | -0.4 | -0.16 | 0.88 |
| Cardio-vascular diseases | 0.015 | 0.013 | 1.3 | 0.56 | 0.57 |
| Respiratory diseases | 0.045 | 0.049 | -1.6 | -0.57 | 0.57 |
| Musculoskeletal diseases | 0.097 | 0.096 | 0.3 | 0.11 | 0.92 |
| Pregnancy and maternity related diseases | 0.141 | 0.138 | 0.6 | 0.22 | 0.82 |
| Diseases of the digestive system | 0.122 | 0.119 | 0.9 | 0.32 | 0.75 |
| Diagnostical examinations | 0.462 | 0.465 | -0.5 | -0.17 | 0.87 |
| Accidents etc. | 0.443 | 0.446 | -0.5 | -0.17 | 0.87 |
| Other diseases | 0.400 | 0.396 | 0.8 | 0.27 | 0.79 |
| **Psychological diagnoses in the past three years** |  |  |  |  |  |
| Organic, including symptomatic, mental disorders | 0.002 | 0.001 | 1.1 | 0.55 | 0.58 |
| Mental and behavioural disorders due to psychoactive substance use | 0.064 | 0.074 | -3.9 | -1.45 | 0.15 |
| Schizophrenia, schizotypal and delusional disorders | 0.017 | 0.020 | -2.0 | -0.96 | 0.34 |
| Mood [affective] disorders | 0.041 | 0.042 | -0.5 | -0.22 | 0.83 |
| Neurotic, stress-related and somatoform disorders | 0.074 | 0.082 | -2.4 | -0.92 | 0.36 |
| Behavioural syndromes associated with physiological disturbances and physical factors | 0.006 | 0.007 | -0.7 | -0.29 | 0.78 |
| Disorders of adult personality and behaviour | 0.027 | 0.033 | -2.6 | -1.15 | 0.25 |
| Mental retardation | 0.005 | 0.006 | -0.3 | -0.12 | 0.91 |
| Disorders of psychological development | 0.015 | 0.015 | -0.5 | -0.19 | 0.85 |
| Behavioural and emotional disorders with onset usually occurring in childhood and adolescence | 0.041 | 0.044 | -1.4 | -0.53 | 0.60 |
| Unspecified mental disorder | 0.011 | 0.011 | -0.4 | -0.19 | 0.85 |
| **Criminal convictions** |  |  |  |  |  |
| violent or sexual crimes | 0.099 | 0.096 | 1.0 | 0.37 | 0.71 |
| Property crime | 0.225 | 0.226 | -0.2 | -0.07 | 0.95 |
| Traffic crime | 0.185 | 0.181 | 1.0 | 0.34 | 0.73 |
| Drug related crime | 0.071 | 0.073 | -0.5 | -0.21 | 0.83 |
| Other crime | 0.085 | 0.087 | -0.7 | -0.26 | 0.80 |
| **Municipality/jobcenter fixed effects** | None significant | | | | |
| **Intervention starting week fixed effects** | None significant | | | | |

**Figure A. Histogram of propensity scores among treated and potential controls.**

**Figure B. Robustness of matching estimator technology.**

**
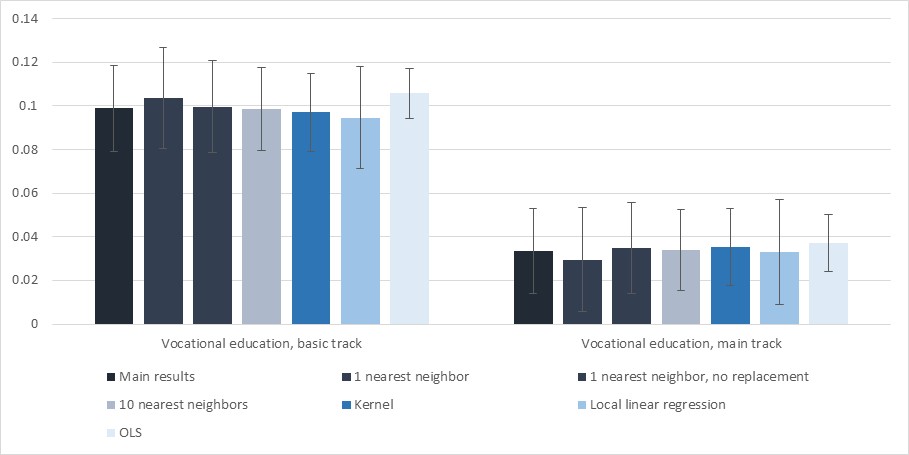
**

Note: Vertical black lines are 95% confidence intervals.
